# Intestinal Mucosal Immune Responses to Novel Oral Poliovirus Vaccine Type 2 in Healthy Newborns

**DOI:** 10.1093/cid/ciaf484

**Published:** 2025-09-05

**Authors:** Audrey Godin, Elizabeth B Brickley, Ruth I Connor, Wendy F Wieland-Alter, Joshua A Weiner, Margaret E Ackerman, John F Modlin, Omar M Sajjad, Minetaro Arita, Chris Gast, Bernardo A Mainou, Khalequ Zaman, Masuma Hoque, Sohel Rana, Ananda S Bandyopadhyay, Peter F Wright

**Affiliations:** Health Equity Action Lab, Department of Infectious Disease Epidemiology and International Health, London School of Hygiene & Tropical Medicine, London, United Kingdom; Health Equity Action Lab, Department of Infectious Disease Epidemiology and International Health, London School of Hygiene & Tropical Medicine, London, United Kingdom; Center for Global Health, Colorado School of Public Health, Aurora, Colorado, USA; Department of Pediatrics, Geisel School of Medicine at Dartmouth, Dartmouth Health, Lebanon, New Hampshire, USA; Department of Microbiology and Immunology, Geisel School of Medicine at Dartmouth, Hanover, New Hampshire, USA; Department of Pediatrics, Geisel School of Medicine at Dartmouth, Dartmouth Health, Lebanon, New Hampshire, USA; Thayer School of Engineering, Dartmouth College, Hanover, New Hampshire, USA; Department of Microbiology and Immunology, Geisel School of Medicine at Dartmouth, Hanover, New Hampshire, USA; Thayer School of Engineering, Dartmouth College, Hanover, New Hampshire, USA; Department of Pediatrics, Geisel School of Medicine at Dartmouth, Dartmouth Health, Lebanon, New Hampshire, USA; Department of Pediatrics, Geisel School of Medicine at Dartmouth, Dartmouth Health, Lebanon, New Hampshire, USA; Department of Virology II, National Institute of Infectious Diseases, Japan Institute for Health Security, Tokyo, Japan; PATH Center for Vaccine Innovation and Access, Seattle, Washington, USA; Division of Viral Diseases, US Centers for Disease Control and Prevention, Atlanta, Georgia, USA; International Centre for Diarrhoeal Disease Research, Dhaka, Bangladesh; International Centre for Diarrhoeal Disease Research, Dhaka, Bangladesh; International Centre for Diarrhoeal Disease Research, Dhaka, Bangladesh; Polio, Global Development, Gates Foundation, Seattle, Washington, USA; Department of Pediatrics, Geisel School of Medicine at Dartmouth, Dartmouth Health, Lebanon, New Hampshire, USA

**Keywords:** polio, vaccine, mucosal immune response, neonatal, antibody

## Abstract

**Background:**

Approximately 1.5 billion doses of novel oral polio vaccine type 2 (nOPV2) have been administered in response to circulating vaccine-derived poliovirus type 2 (cVDPV2) outbreaks since 2021. Although infants are eligible to receive the vaccine from birth, the induction of intestinal mucosal immunity by nOPV2 in newborns has not been directly evaluated.

**Methods:**

In a randomized, placebo-controlled, phase 2 clinical trial in Bangladesh (2020–2021), 215 healthy newborns received 2 doses of either nOPV2 (n = 110) or placebo (sucrose; n = 105), at birth (0–3 days) and 4 weeks later. Intestinal mucosal antibody responses were assessed by measuring poliovirus type 2 (PV2)-specific neutralizing activity and immunoglobulin (Ig)A levels in stool collected biweekly from birth to 8-weeks.

**Results:**

Newborns vaccinated with 2 doses of nOPV2 had strong intestinal mucosal antibody responses that differed significantly from the placebo group (*P* < .0001 for PV2-specific neutralization from 2 weeks onward and *P* ≤ .007 for PV2-specific IgA from 4 weeks onward). Positive PV2-specific neutralization in stool (titers ≥16) was detected in 51.8% (57/110) of nOPV2-vaccinated newborns at 4 weeks and 90.0% (99/110) at 8 weeks (4 weeks after the second dose). Notably, PV2-specific antibody titers following the second dose were very similar for newborns who did and did not have first dose responses (*P* = .67 for neutralization and *P* = .38 for IgA at 8 weeks).

**Conclusions:**

Vaccination with 2 doses of nOPV2 in neonates induced strong intestinal mucosal antibody responses. In cVDPV2 outbreak settings, neonatal administration of nOPV2 may be a strategy to enhance population-level intestinal mucosal immunity.

## Teaser

This study shows that novel oral polio vaccine type 2 (nOPV2) induced strong type 2-specific intestinal mucosal antibody responses after 2 doses, at birth and 4 weeks later, supporting the WHO recommendation of using nOPV2 from birth in outbreak response.

Circulating vaccine-derived type 2 polioviruses (cVDPV2s) are currently the leading cause of poliomyelitis and threaten global poliovirus (PV) eradication efforts [1]. In response to cVDPV2 outbreaks, approximately 1.5 billion doses of novel oral poliovirus vaccine type 2 (nOPV2), which is less likely to acquire genetic mutations linked to neurovirulence than the historically used Sabin oral PV vaccine type 2, have been administered since 2021 [2–4]. In infants (18–22 weeks), children (1–5 years), and adults (18–50 years), nOPV2 has been shown to be safe, well-tolerated, and capable of inducing both serum immunity, which confers individual protection against paralytic poliomyelitis, and intestinal mucosal immunity, which reduces PV replication and onward fecal-oral transmission [5–13].

Although infants are eligible to receive nOPV2 from birth, the induction of intestinal mucosal immunity by nOPV2 in newborns has not been directly evaluated. Intestinal mucosal immunity to PV is typically inferred by a decrease of vaccine virus shedding in stools after challenge with oral polio vaccine (OPV). In the absence of a challenge, PV type-specific intestinal mucosal immune responses can be evaluated by measuring neutralizing activity and immunoglobulin (Ig) A responses, which have been shown to be positively correlated with each other [8, 14, 15] and inversely correlated with vaccine virus shedding after homologous OPV challenge [16]. Using paired stool and serum samples from 215 healthy newborns participating in a phase 2 clinical trial of nOPV2 in Bangladesh [9], we evaluated the induction of PV type 2 (PV2)-specific neutralizing activity and IgA levels in stool, as a proxy for mucosal immunity, in newborns vaccinated with 2 doses of nOPV2 (at birth and 4 weeks of age) compared to a placebo arm and investigated the influence of maternally derived antibodies in the detection of intestinal mucosal responses during the neonatal period.

## METHODS

### Study Design and Participants

Intestinal mucosal antibody responses were measured after 1 and 2 doses of nOPV2 in newborns included in a placebo-controlled, randomized, double-blinded phase 2 clinical trial (NCT04693286; N = 330) conducted at the Matlab Health Research Center, Chandpur, Bangladesh, between September 2020 and August 2021. The study design, eligibility criteria, interventions, primary safety, and serum immunogenicity outcomes have been reported previously [9]. Eligible participants included healthy, singleton newborns born at ≥37 weeks of gestation. The main exclusion criteria were: (1) diagnosis or suspicion of immunodeficiency disorder in the newborn or immediate family member, (2) contraindication for venipuncture, (3) infection or illness requiring hospitalization, (4) vomiting or intolerance to liquids <24 h before enrollment, and/or (5) prior receipt of any PV and/or rotavirus vaccine.

Participants received 2 doses of nOPV2 (10^5.0+/−0.5^ 50% cell culture infectious dose [CCID_50_], PT Bio Farma, Bandung, Indonesia) or placebo (sucrose in Basal Medium Eagle and buffer, PT Bio Farma) as 2 oral drops (0.1 mL) at birth (0–3 days) and 4 weeks later. Serum was collected at birth, weeks 4 and 8; stool was collected every 2 weeks from birth. Samples collected at week 4 were obtained prior to administration of the second dose. Samples were shipped frozen to the Polio and Picornavirus Branch of the Division of Viral Diseases, Centers for Disease Control and Prevention (CDC, Atlanta, GA, USA), where serum samples were analyzed using a PV serotype-specific microneutralization assay (PMID 26983734) [17] and stool samples were qualitatively evaluated for the presence of PV serotype-specific shedding using real-time reverse transcription polymerase chain reaction (RT-PCR) and, if PV2-positive, quantitatively measured as CCID_50_ per g of stool as previously reported [9].

### Laboratory Procedures

Intestinal mucosal antibody responses were evaluated in stool from a subset of participants selected for having a complete set of stools across visits (110 vaccine recipients and 105 placebo recipients). Samples were aliquoted and sent frozen to the Geisel School of Medicine at Dartmouth College (Hanover, NH, USA). PV1-, PV2-, and PV3-specific neutralization titers in stool were reported as the reciprocal of the limiting dilution needed to achieve 60% neutralization of luciferase-expressing wild-type-derived polio pseudo-viruses [18]. Neutralization titers <4 (lower limit of detection) were recorded as 2. In the nOPV2 group, samples with PV2-specific titers >512 were serially diluted further, up to 16,384, to capture the highest end-point titers. Total IgA concentrations (µg/mL) and PV1-, PV2-, PV3-specific IgA and IgG levels (expressed as median fluorescence intensity [MFI] and, if negative after background signal subtraction, recorded as half of the lowest observed homotypic MFI) in stool were quantified using a multiplex assay developed by coupling monovalent inactivated PV vaccines to fluorescently coded magnetic microspheres, as described previously [14]. Lactoferrin (ng/mL) in stool was measured at birth using a commercially available enzyme-linked immunosorbent assay (ELISA) per manufacturer's instructions (Lactoferrin Scan® TechLab, Inc., Blacksburg, VA, USA) as a biomarker of breastmilk consumption.

### Statistical Analyses

Differences in the distribution of PV serotype-specific neutralization, IgA, and IgG levels were assessed using Mann–Whitney *U* tests. Differences in the proportion of positive neutralizing activity (defined as neutralization titers ≥16) and detectable vaccine viral shedding (PV2-positive RT-PCR) in stool at each visit and/or ever (in at least 1 visit) were assessed using Pearson's χ^2^ tests. To evaluate the effect of the second dose of nOPV2 on intestinal mucosal antibody responses, we categorized participants as birth dose responders (neutralization titers in stool ≥16 at 2 and/or 4 weeks [before the second dose]) or nonresponders (titers <16 at 2 and 4 weeks). To investigate if placentally transferred maternal antibodies interfered with the detection of intestinal mucosal responses after the birth dose, we calculated the odds ratios for the association of (1) positive neutralization and (2) PV2-positive RT-PCR in stool at 2 and/or 4 weeks with the magnitude of serum neutralization at birth in tertiles. As a sensitivity analysis for the threshold of positive neutralization, we repeated the analyses using titers ≥4 (detectable). As a post hoc analysis to determine the influence of breastfeeding on stool IgA levels observed at baseline, we computed the Spearman's rank correlations between lactoferrin, total IgA, and PV serotype-specific IgA.

All *P* values are from 2-sided tests. All analyses were performed using Stata, v17.0, and R, v4.2.0.

## ETHICS

The International Centre for Diarrhoeal Disease Research, Bangladesh, provided ethical approval for the parent trial [9]. All newborns’ parents/guardians provided written informed consent before enrollment, including the use of samples for further polio-related studies. Dartmouth-Hitchcock Institutional Review Board approved the deidentified samples analysis (#02002410).

## RESULTS

We evaluated PV1-, PV2-, and PV3-specific intestinal mucosal antibody responses in 215 newborns (50.2% female), representing 65% of participants in the parent trial. At baseline, infants in the nOPV2 and placebo groups were similar in terms of sex, age, birth weight, breastfeeding status, and Bacillus Calmette–Guérin (BCG) vaccination (Supplementary Table 1). Most newborns were exclusively breastfed at birth (99.5% [214/215]) and at least partially breastfed at 4 weeks (99.1% [213/215]). The median (interquartile range, IQR) total IgA measured in stools was 0.5 (0.2–35.1) µg/mL at baseline and ranged from 46.0 (27.8–75.0) to 61.3 (39.8–92.8) µg/mL during subsequent visits. At baseline, positive neutralizing activity (titers ≥16) in stool was detected in 1.8% (2/110) of participants in the nOPV2 group and 1.9% (2/105) in the placebo group (median [IQR] titers: 2 [2–2]).

**Figure 1.**
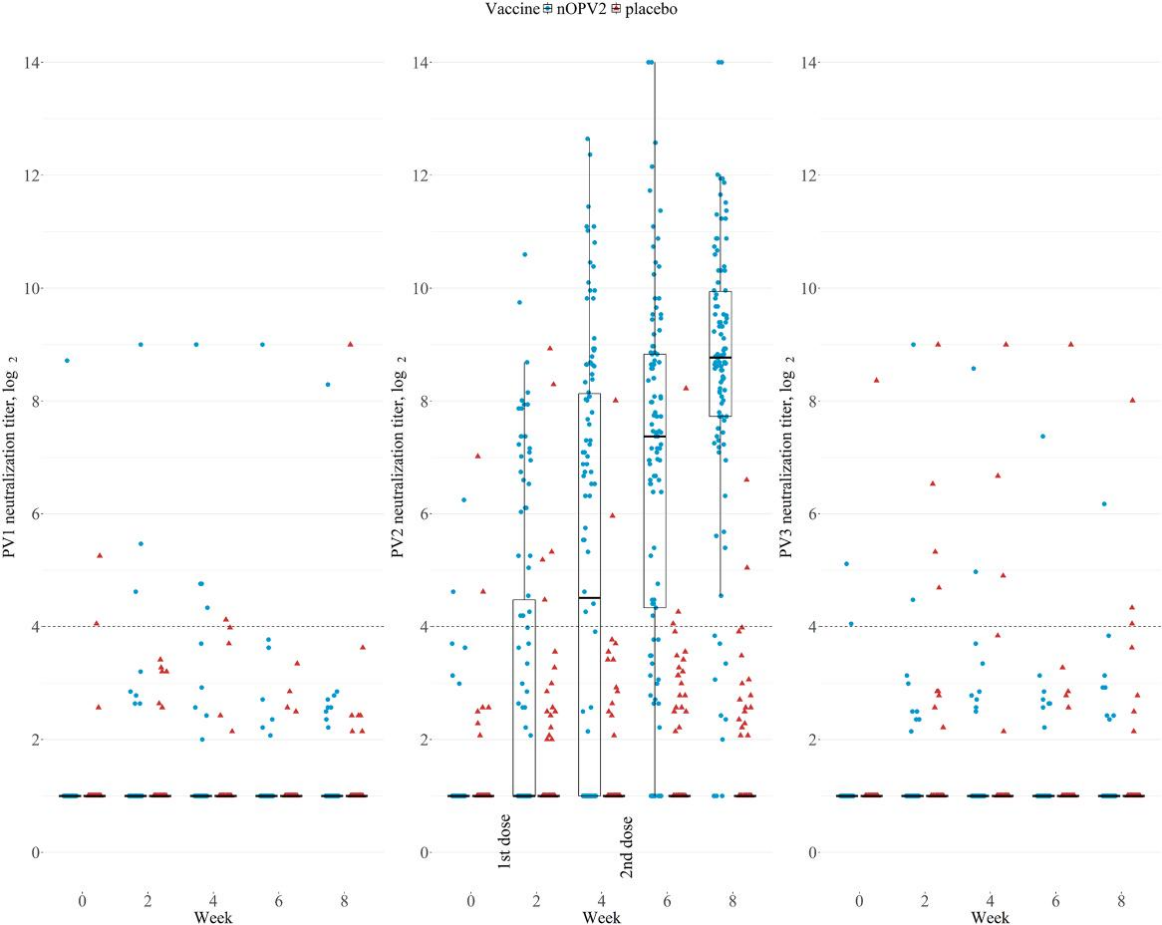
Distribution of poliovirus serotype-specific (PV1, PV2, and PV3) log_2_ neutralization titers in stool after vaccination with the novel oral polio vaccine type 2 (nOPV2) or placebo. Samples at weeks 0 and 4 were collected before vaccination with nOPV2 or placebo. Colors and shapes indicate the vaccine received. Horizontal lines from the boxplots indicate the median; box, and whisker indicate the interquartile range (IQR) and 1.5*IQR. The dashed lines indicate the limit of positivity (titers ≥16). Neutralization titers <4 (lower limit of detection) were recorded as 2 (log_2_ titers = 1). The upper limit of detection of neutralization titers was set at 512 (log_2_ titers = 9) for PV1 and PV3 and 16,384 (log_2_ titers = 14) for PV2.

**Figure 2.**
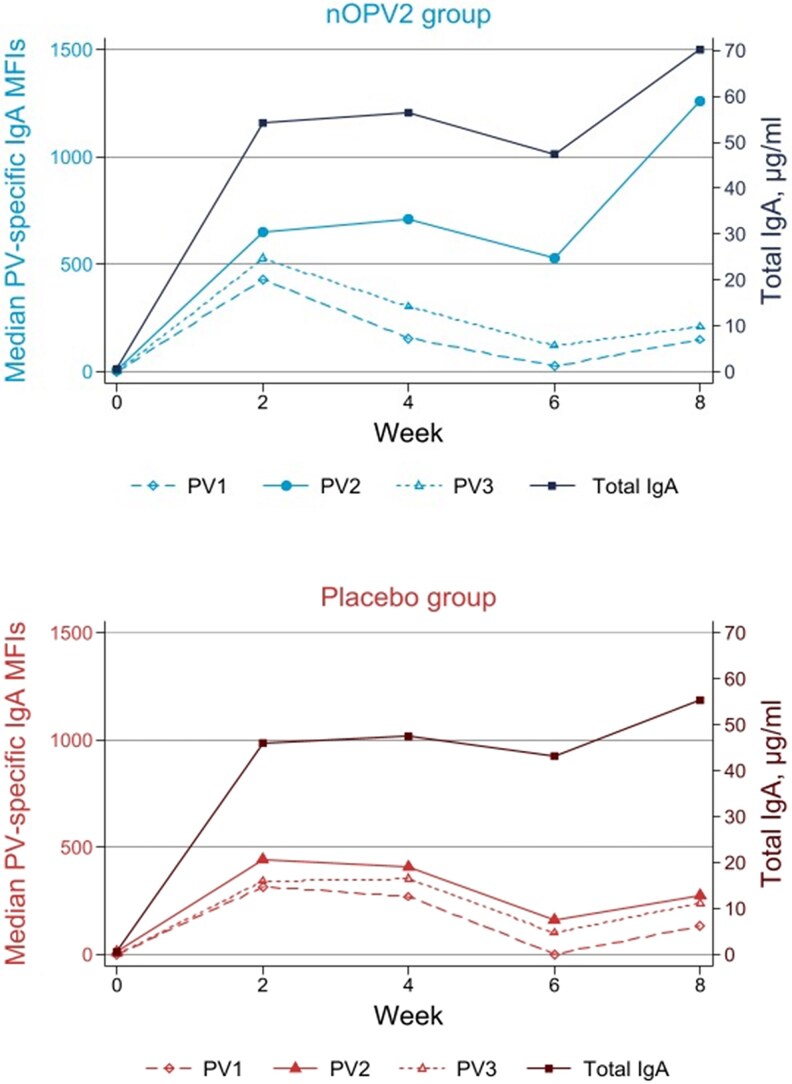
Median total immunoglobulin A (IgA, µg/mL) and poliovirus serotype-specific (PV1, PV2, and PV3) median fluorescence intensities (MFIs) IgA in stool after vaccination with the novel oral polio vaccine type 2 (nOPV2) or placebo.

The nOPV2 birth dose induced positive neutralizing activity in stool in 28.2% (31/110) participants by 2 weeks (median [IQR] titers: 2 [2–23.4]) and 51.8% (57/110) at 4 weeks (median [IQR] titers: 22.8 [2–284.3]); compared with 4.8% (5/105) and 1.9% (2/105) at weeks 2 and 4 respectively (median [IQR] titers: 2 [2–2]) in the placebo group (Table 1 and Figure 1). PV2-specific IgA levels in stool rose progressively after the nOPV2 birth dose to a median (IQR) MFI of 711.6 (228.6–1599.6) at 4 weeks (Table 1 and Figure 2). In contrast, PV2-specific IgA levels in the placebo group and PV1- and PV3-specific IgA levels in all participants remained low (Figure 2). Stool PV2-specific IgG levels were largely undetectable in follow-up (Table 1).

**Table 1.** Poliovirus Type 2 (PV2)–Specific Intestinal Mucosal Responses Following Administration of Novel Type 2 Oral Poliovirus Vaccine (nOPV2) or Placebo

In stool samples	Week	nOPV2 (N = 110^a^) n/N (%) or median (IQR)	Placebo (N = 105) n/N (%) or median (IQR)	*P* Value
**Positive PV2 neutralizing activity**	Baseline	2/110 (1.8%)	2/105 (1.9%)	.96
	2	31/110 (28.2%)	5/105 (4.8%)	**<.0001**
	4	57/110 (51.8%)	2/105 (1.9%)	**<.0001**
	6	83/109 (76.2%)	3/105 (2.9%)	**<.0001**
	8	99/110 (90.0%)	2/105 (1.9%)	**<.0001**
**PV2 neutralization titer**	Baseline	2 (2–2)	2 (2–2)	.75
	2	2 (2–23.4)	2 (2–2)	**<.0001**
	4	22.8 (2–284.3)	2 (2–2)	**<.0001**
	6	165.9 (20.2–454.5)	2 (2–2)	**<.0001**
	8	437.4 (211.9–995.7)	2 (2–2)	**<.0001**
**PV2 IgA MFI**	Baseline	9.1 (0.1–49.1)	11.1 (0.1–52.1)	.72
	2	651.1 (289.1–1550.1)	445.1 (153.1–1191.6)	**.02**
	4	711.6 (228.6–1599.6)	408.1 (199.1–841.1)	**.007**
	6	528.1 (224.1–1118.6)	162.1 (66.1–344.1)	**<.0001**
	8	1260.4 (737.1–2154.1)	277.1 (132.6–621.1)	**<.0001**
**Total IgA (µg/mL)**	Baseline	0.5 (0.2–52.5)	0.5 (0.2–17.6)	.63
	2	54.2 (35.3–113.1)	46.0 (30.7–66.8)	.07
	4	56.5 (38.8–108.0)	47.4 (34.7–69.8)	**.02**
	6	47.5 (32.8–77.4)	43.2 (21.1–68.0)	.08
	8	70.2 (43.3–107.5)	55.4 (36.7–82.6)	.08
**PV2 IgG MFI**	Baseline	0.0 (0.0–0.0)	0.0 (0.0–0.0)	.71
	2	0.0 (0.0–0.0)	0.0 (0.0–0.0)	.98
	4	0.0 (0.0–0.0)	0.0 (0.0–0.0)	.54
	6	0.0 (0.0–172.1)	0.0 (0.0–79.6)	.16
	8	0.0 (0.0–205.1)	0.0 (0.0–100.1)	.24
**PV2-positive RT-PCR**	2	57/110 (51.9%)	1/105 (1.0%)	**<.0001**
	4	42/110 (38.2%)	0/105 (0%)	**<.0001**
	6	69/110 (62.7%)	2/105 (1.9%)	**<.0001**
	8	42/110 (38.2%)	0/105 (0%)	**<.0001**
**Ever detectable PV2-positive RT-PCR**	2–8	105/110 (95.5%)	3/105 (2.9%)	**<.0001**

*P* values in bold are ≤.05.

*P* values are from Pearson's χ^2^ or Mann–Whitney *U* tests. Neutralization titers ≥16 were considered a positive neutralizing activity. Abbreviations: IgA, Immunoglobulin A; MFI, median fluorescence intensity; IQR, interquartile range; RT-PCR, reverse transcription polymerase chain reaction.

^a^N = 109 at baseline for total IgA, in week 4 for the PV2 IgA, and in week 6 for all measurements in the nOPV2 group.

We compared the 59 (53.6%) nOPV2 birth dose responders (with PV2-specific neutralization titers ≥16 at 2 and/or 4 weeks) to the 51 (46.4%) birth dose nonresponders. At 2 weeks, 93.2% (55/59) of responders had PCR-detectable PV2 shedding in stool compared with 3.9% (2/51) of nonresponders (Table 2). In contrast, 92.2% (47/51) of the nonresponders had detectable PV2 shedding in stool 2 weeks after the second dose (week 6). Among birth dose responders, 37.3% remained RT-PCR PV2-positive at 6 weeks, reflecting a combination of ongoing birth dose shedding and virus shedding from the second dose. As previously reported, the magnitude of shedding after the first and second doses of nOPV2 was remarkably low, with a median log_10_ CCID_50_ of 2.75 (the lower limit of detection) [9] and did not differ between birth dose responders and nonresponders (Supplementary Figure 1). At 8 weeks, most birth dose nonresponders (90.2% [46/51]) had positive PV2-specific neutralizing activity, and PV2-specific IgA levels and neutralization titers in stool were comparable between the birth dose responders and nonresponders (*P* ≥ .38, Table 2; Supplementary Figure 2).

**Table 2.** Stool and Serum Poliovirus Type 2 (PV2)–Specific Responses Following Administration of the Novel Type 2 Oral Poliovirus Vaccine (nOPV2) Second Dose in Responders or Nonresponders to the First Dose

	Week	Birth Dose Responder (N = 59^a^)	Birth Dose Nonresponder (N = 51^b^)	*P* Value
		n/N(%) or median (IQR)	n/N(%) or median (IQR)	
In stool samples				
**Positive PV2 neutralizing activity**	Baseline	2/59 (3.4%)	0/51 (0%)	.19
	2	31/59 (52.5%)	0/51 (0%)	…^c^
	4	57/59 (96.6%)	0/51 (0%)	…^c^
	6	52/59 (88.1%)	31/50 (62.0%)	**.001**
	8	53/59 (89.8%)	46/51 (90.2%)	.95
**PV2 neutralization titer**	Baseline	2 (2–2)	2 (2–2)	.90
	2	18.3 (2–136.3)	2 (2–2)	…^c^
	4	270.7 (106.7–553.0)	2 (2–2)	…^c^
	6	400.7 (143.2–902.7)	32.1 (6.5–165.9)	**<.0001**
	8	412.1 (182.1–1098.3)	442.0 (211.9–818.5)	.67
**PV2 IgA MFI**	Baseline	21.1 (0.1–52.1)	4.1 (0.1–49.1)	.32
	2	759.6 (391.1–1550.1)	398.1 (232.1–1735.6)	.29
	4	1198.4 (546.1–2077.1)	345.1 (134.1–797.1)	**<.0001**
	6	902.1 (443.1–1481.1)	251.9 (99.6–604.6)	**<.0001**
	8	1187.1 (625.6–2131.1)	1381.6 (737.1–2280.6)	.38
**Total IgA (µg/mL)**	Baseline	0.7 (0.2–53.9)	0.5 (0.2–45.3)	.47
	2	51.1 (35.6–113.1)	58.7 (33.7–116.0)	.75
	4	62.3 (42.1–127.7)	51.9 (38.6–77.7)	**.03**
	6	43.5 (27.6–71.3)	54.8 (35.3–88.8)	**.05**
	8	65.3 (40.2–97.0)	72.4 (43.4–121.5)	.22
**PV2-positive RT-PCR**	2	55/59 (93.2%)	2/51 (3.9%)	**<.0001**
	4	40/59 (67.8%)	2/51 (3.9%)	**<.0001**
	6	22/59 (37.3%)	47/51 (92.2%)	**<.0001**
	8	11/59 (18.6%)	31/51 (60.8%)	**<.0001**
**Ever detectable PV2-positive RT-PCR**	2–8	58/59 (98.3%)	47/51 (92.2%)	.12
**In serum samples**				
**PV2 neutralization titer, log2**	Baseline	5.2 (3.8–6.8)	5.8 (4.8–7.8)	**.05**
	4	9.2 (7.5–10.5)	4.5 (3.5–6.8)	**<.0001**
	8	10.2 (8.5–10.5)	10.2 (9.2–10.5)	.41

*P* values in bold are ≤.05.

*P* values are from Pearson's χ^2^ or Mann–Whitney *U* tests. Responders are participants with stool neutralization titers ≥16 (positive neutralizing activity) at week 2 and/or week 4 (before the second dose). Abbreviations: IgA, Immunoglobulin A; MFI, median fluorescence intensity; IQR, interquartile range; RT-PCR, reverse transcription polymerase chain reaction.

^a^N = 58 at week 4 for IgA and at baseline for total IgA in stool; at week 8 for neutralization in serum.

^b^N = 50 at week 6 for all measurements in stool; at week 8 for neutralization in serum.

^c^No *P* values were calculated as the groups were defined by the absence of neutralizing activity at weeks 2 and 4.

Birth dose responders and nonresponders did not differ in terms of baseline characteristics of sex, weight, breastfeeding, and BCG receipt (Supplementary Table 2). However, 41.5% (17/41) of the newborns who had low serum neutralization titers at baseline (lowest tertile) had positive neutralizing activity in stool 2 weeks after receiving a birth dose of nOPV2 as compared to 20.0% (7/35) of those with high baseline serum neutralization (highest tertile; Table 3). Relative to newborns with high serum neutralization titers at baseline, those with low serum neutralization titers had 2.8 (95% CI: 1.0–8.0, *P* = .05) times the odds of having positive neutralizing activity in stool and 2.6 (95% CI: 1.0–6.5, *P* = .05) times the odds of being RT-PCR PV2-positive at 2 weeks (Table 3). There was no association between baseline serum neutralization titers and positive neutralizing activity in stool at 4 weeks. In sensitivity analysis using detectable stool neutralization titers (≥4), those with low serum neutralization titers had 3.9 (95% CI: 1.4–10.6, *P* = .008) times the odds of having positive neutralizing activity in stool (Supplementary Table 3).

**Table 3.** Association of Poliovirus Type 2 (PV2)-specific Positive Neutralizing Activity and PV2-positive RT-PCR in Stool 2 and 4 Weeks After Receiving the First Dose of Novel Type 2 Oral Poliovirus Vaccine (nOPV2) With the Magnitude of PV2-specific Serum Neutralization Titers at Baseline (in Tertiles)

	Positive Neutralizing Activity in Stool 2 Weeks After first Dose of nOPV2 (n = 31/110)				Positive Neutralizing Activity in Stool 4 Weeks After first Dose of nOPV2 (n = 57/110)				PV2-positive RT-PCR in Stool 2 Weeks After first Dose Of nOPV2 (n = 57/110)			
Baseline log_2_ Neutralization Titers in Serum	n/N (%)	OR	95% CI	*P Value*	n/N (%)	OR	95% CI	*P Value*	n/N (%)	OR	95% CI	*P* Value
High (6.8–10.5)	7/35 (20.0%)	Ref	Ref	…	17/35 (48.6%)	Ref	Ref	…	15/35 (42.9%)	Ref	Ref	…
Medium (5.1–6.5)	7/34 (20.6%)	1.0	0.3–3.4	0.95	14/34 (41.2%)	0.7	0.3–1.9	0.54	15/34 (44.1%)	1.1	0.4–2.7	.92
Low (<2.5–4.8)	17/41 (41.5%)	2.8	1.0–8.0	**0.05**	26/41 (63.4%)	1.8	0.7–4.6	0.19	27/41 (65.9%)	2.6	1.0–6.5	**.05**

*P* values in bold are ≤.05.

*P* values are from Pearson's χ^2^ tests. Neutralization titers ≥16 were considered a positive neutralizing activity. Abbreviation: RT-PCR, reverse transcription polymerase chain reaction; OR, odds ratio; 95% CI, 95% confidence interval.

At 8 weeks, 90.0% (99/110) of all nOPV2 recipients had positive PV2-specific neutralizing activity in stool (median [IQR] titers: 437.4 [211.9–995.7], Table 1). In contrast, few newborns had positive PV1- or PV3-specific neutralizing activity in stool during follow-up, indicating little circulation of the vaccine virus or serotype cross-reactivity in the assay (Figure 1; Supplementary Table 4). In a sensitivity analysis, 106/110 (96.4%) participants in the nOPV2 group and 17/105 (16.2%) in the placebo group had detectable neutralizing activity (titers ≥4) at 8 weeks (Supplementary Table 5). Overall, the PV2-specific neutralization titers and IgA MFIs in stool in the nOPV2 group were similar between males and females and between the participants who did and did not receive a BCG vaccine at birth (Supplementary Table 6).

In the posthoc analysis of lactoferrin, we observed strong positive correlations between total IgA, PV serotype-specific IgA MFIs, and lactoferrin concentrations in the newborns’ baseline stool samples (Spearman's rho ≥0.41, *P* < .0001 for all, Supplementary Figure 3).

## DISCUSSION

Our findings provide evidence that vaccination with 2 doses of nOPV2, administered at birth and 4 weeks, induces strong PV2-specific intestinal neutralizing and binding antibodies in stool. While half of the vaccine recipients had functional neutralizing activity after the birth dose, 90% of all nOPV2 recipients had positive PV2-specific neutralizing activity in stool following the second dose. Overall, our findings support the WHO recommendation that nOPV2 may be used in cVDPV2 outbreak responses from birth [19]; however, a second dose might be necessary to effectively elicit intestinal mucosal antibody responses in most newborns.

Although it has been reported that neonatal immunization with some vaccines induces weaker and shorter serum immune responses [20, 21], the median PV2-specific stool neutralization titers observed after 2 doses of nOPV2 (median of 437 at 8 weeks) were similar to the peak neutralization titers reported in OPV2-naïve infants (28 weeks of age) in Chile after 1 dose of mOPV2 (median ranging from 362 to 388 across trial arms) [15] and higher than the peak neutralization titers reported in OPV2-naïve toddlers (18-months-old) in Panama after 1 dose of nOPV2 (median of 84) and OPV2-naïve adults in Belgium after 1 dose of nOPV2 (median of 2), in studies using the same stool collection method and immunoassays [8, 22]. Some variability between studies assessing intestinal mucosal immunity, measured as odds of stool shedding after vaccine challenge, in older children, has been reported, likely owing to heterogeneity in sample collections, analyses, or vaccine schedules [23]. Similarly, differences in responses across the serotypes have been shown, particularly to type 3 after a single dose of trivalent OPV [23], and our results are not generalizable to potential concomitant use with other polio vaccines (eg, bivalent OPV [bOPV] birth doses). Large variability in serum immunogenicity after an OPV birth dose has been observed [24], and further research is needed to determine whether the difference in the magnitude of neutralization responses in stool is due to maternal immunity, the age of vaccine recipients, number of doses administered, or vaccine setting.

The PV2-specific neutralization in stools was slower to peak after receipt of nOPV2 at both birth and 4 weeks than previously observed in older individuals. We have previously reported that PV2-specific neutralization and IgA MFIs in stool peaked by 2 weeks after mOPV2 vaccination in OPV2-naïve infants (aged 18–32 weeks) in Latin America [15, 16]. Only 38% of nOPV2 recipients in our study had detectable neutralization (titers ≥4) 2 weeks after the birth dose, and 55% at week 4. In contrast, we previously observed detectable PV2-specific neutralization in stool in 94–98% of OPV2-naïve infants (aged 18–22 weeks) 2 weeks after mOPV2 challenge [25] and 82% of infants 2 weeks after 1 dose of nOPV2 [8]. Further research is necessary to understand temporal differences in the induction of mucosal immunity to nOPV2 received at birth versus later infancy.

The absence of positive PV2-specific neutralizing activity in stool in half the nOPV2 recipients after the birth dose warrants consideration. The lack of birth dose neutralization responses and the delayed peak in antibodies compared to older infants may reflect lower replication of PV in the neonatal gut, interference of maternal antibodies (serum IgG passively transferred through the placenta and secretory IgA in the breastmilk), or age-related differences in rates of B cell affinity maturation. While critical to protecting newborns, maternal antibodies are known to interfere with early vaccine responses [21, 26], including those to PV vaccines [27–29]. High maternal antibodies measured in cord blood, serum, or colostrum have been associated with lower responses to a birth dose of OPV as measured by stool viral excretion or serum antibody response [20, 28–30]. In our study, high serum neutralization at baseline was associated with lower odds of detectable neutralizing activity in stool at 2 weeks, but not at 4 weeks. Based on the lactoferrin correlations, we hypothesize that the low levels of type 2-specific IgA observed in the vaccine and placebo arms at baseline may be breastmilk-derived. However, clear differences in type 2-specific IgA levels emerged between the vaccine and placebo arms from 2 weeks onwards.

Alternatively, immunologic priming by nOPV2 birth dose might explain why we observed similar functional activity and binding antibody levels between birth dose responders and nonresponders at 8 weeks. It has been suggested that priming may occur, via B cell memory, based on observations of robust antibody responses after a second vaccine dose in early life [21, 31]. However, our findings are not consistent with an anamnestic response, as we observed similar magnitudes of neutralizing activity 2 weeks after both the first dose in responders and the second dose in nonresponders. Additionally, only 3.9% of birth dose nonresponders had detectable virus shedding after the first dose, while 92.2% shed after the second dose, suggesting that the first dose of the vaccine might have had a limited “take” due to inefficient replication of nOPV2 in the neonatal intestinal mucosa. The swifter response in the nonresponders after the second dose as compared as the responder after the first dose might be explained by interference of maternal antibodies or reflect a more mature immune system. Notably, the vaccine virus shedding titers in stool (measurable by cell culture) at week 2 were very low in the newborn, as reported by the parent study [9], although a previous study in older infants suggests that shedding peaks 7 days after vaccination [7]. While mucosal immunity in the neonatal intestine typically develops rapidly after birth, concurrent with the gut microbiota [32], failure to induce detectable enteric IgA after 1 dose of nOPV2 in some newborns may also be explained by an age-dependent lack of endogenous IgA production in the intestine at birth [33]. While the assessment of the kinetics and duration of mucosal antibody responses after 1 birth dose of nOPV2 is limited in our study by the systematic administration of a second dose 4 weeks later, studies are needed to compare a 1-dose neonatal schedule to a 2-dose schedule. As IPV is known to boost mucosal antibody responses in previously OPV-exposed children [34–36], the intestinal mucosal responses in nOPV2 vaccinated newborns should be further evaluated after IPV administered subsequently per national immunization programs.

Neonatal nOPV2 administration may be a strategy to enhance population-level intestinal mucosal immunity in cVDPV2 outbreak settings. Vaccinating newborns offers logistical and clinical advantages, as they are more likely to have increased contact with the healthcare system near birth, less likely to be exposed to other enteric infections known to diminish the effectiveness of OPV in older infants [26, 37], and less likely to develop vaccine-associated paralytic poliomyelitis because of maternally protective serum antibody [24]. We hypothesize that neonatal vaccination may also mitigate risks of a susceptibility window that could occur as maternal antibodies wane and before routine immunization is completed and of onward transmission of the virus, as newborns appear to shed vaccine virus at lower magnitude than older infants. This study provides preliminary evidence that the magnitude of intestinal mucosal neutralizing activity induced by neonatal nOPV2 administration is similar or higher to those observed with mOPV2 or nOPV2 challenges in later life. Further research on neonatal PV vaccination is warranted to understand the optimal number and timing of doses, the duration of protective immunity, and potential effect modifiers including formula feeding, prematurity, or concomitant administration of bOPV.

## Supplementary Material

ciaf484_Supplementary_Data

## Supplementary Data

Supplementary materials are available at *Clinical Infectious Diseases* online. Consisting of data provided by the authors to benefit the reader, the posted materials are not copyedited and are the sole responsibility of the authors, so questions or comments should be addressed to the corresponding author.
