# Supplementary material for: Intestinal Mucosal Immune Responses to Novel Oral Poliovirus Vaccine Type 2 in Healthy Newborns
Source: Clin Infect Dis. 2025 Sep 5;82(2):e352–60. doi: 10.1093/cid/ciaf484 (PMC13017628; doi:10.1093/cid/ciaf484)
Supplement: ciaf484_Supplementary_Data [file ciaf484_supplementary_data.docx]

**Supplementary Table 1. Baseline characteristics of the study participants.** Abbreviations: nOPV2, novel oral polio vaccine type 2; Sd, standard deviation; BCG, Bacillus Calmette-Guérin.

* All breastfed newborns were reported to be exclusively breastfed at birth, and partially breastfed at week 4

|  | nOPV2 (N=110)  n/N (%) \| mean (sd) | Placebo (N=105)  n/N (%) \| mean (sd) |
| --- | --- | --- |
| Sex |  |  |
| Male | 53/110 (48.2%) | 54/105 (51.4%) |
| Female | 57/110 (51.8%) | 51/105 (48.6%) |
| Age (days) | 1.1 (0.7) | 1.1 (0.6) |
| Weight (kg) | 2.9 (0.3) | 2.8 (0.3) |
| Breastfeeding* |  |  |
| At baseline | 109/110 (99.1%) | 105/105 (100%) |
| At week 4 visit | 109/110 (99.1%) | 104/105 (99.1%) |
| Received BCG vaccine |  |  |
| At birth | 44/110 (40.0%) | 49/105 (46.7%) |
| Between birth and week 4 visit | 26/110 (23.6%) | 21/105 (20.0%) |

**Supplementary Figure 1. Poliovirus type 2 specific stool shedding measured as 50% cell culture infectious dose (CCID_50_) per gram of stool after vaccination with the novel oral polio vaccine type 2 (nOPV2) in birth dose responders and non-responders.** Responders are participants with stool neutralization titers ≥16 at week 2 and/or week 4.

**
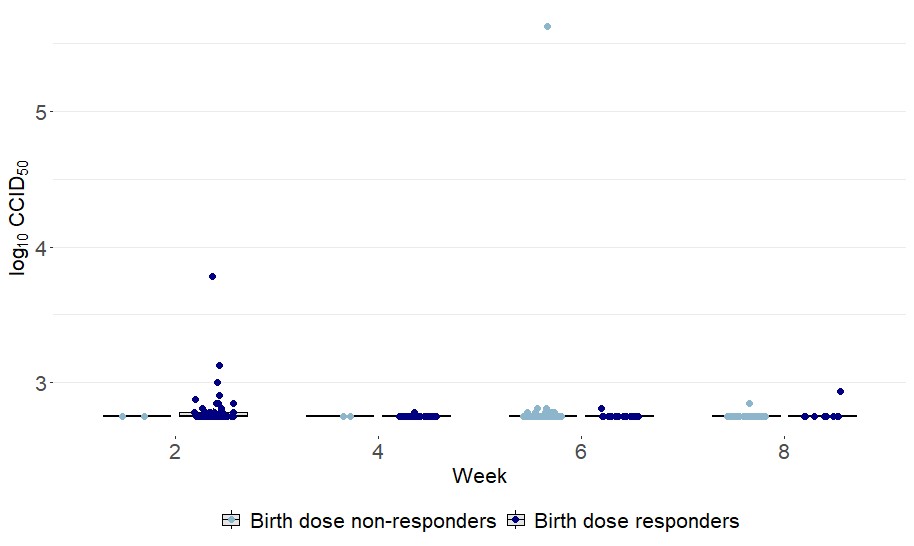
**

**Supplementary Table 2. Baseline characteristics of the responders and non-responders to the first dose of nOPV2 vaccine.** *P* values are from *t*-tests and Pearson’s chi-squared tests. Responders are participants with stool neutralization titers ≥16 at week 2 and/or week 4. Abbreviations: nOPV2, novel oral polio vaccine type 2; Sd, standard deviation; BCG, Bacillus Calmette-Guérin.

|  | Birth dose responder (N=59)  n/N (%) \| mean (sd) | Birth dose non-responder (N=51)  n/N (%) \| mean (sd) | *P* value |
| --- | --- | --- | --- |
| Sex male | 26/59 (44.1%) | 27/51 (53.0%) | 0.35 |
| Age (days) | 1.2 (0.7) | 0.9 (0.7) | **0.04** |
| Weight (kg) | 2.9 (0.3) | 3.0 (0.4) | 0.20 |
| Breastfeeding | 59/59 (100%) | 50/51 (98.0%) | 0.28 |
| Received BCG vaccine | 24/59 (40.7%) | 20/51 (39.2%) | 0.88 |

**Supplementary Figure 2. Distribution of poliovirus type 2 (PV2)-specific log_2_ neutralization titers, median total immunoglobulin A (IgA, µg/ml), and PV2-specific median fluorescence intensities (MFIs) IgA in stool samples after vaccination with the novel oral polio vaccine type 2 (nOPV2) in birth dose responders and non-responders.** Responders are participants with stool neutralization titers ≥16 at week 2 and/or week 4. On the left plot: the dashed lines indicate the limit of positivity for the neutralization (titers ≥16); horizontal lines indicate the median; box and whisker indicate the interquartile range (IQR) and 1.5*IQR.

**Supplementary Table 3. Association of poliovirus type 2 (PV2)-specific detectable neutralization titers in stool two and four weeks after receiving the first dose of novel type 2 oral poliovirus vaccine (nOPV2) with the magnitude of PV2-specific neutralization in serum at baseline.** *P* values are from Pearson’s chi-squared tests. Neutralization titers ≥4 were considered detectable. Abbreviations: OR, odds ratio; 95% CI, 95% confidence interval.

|  | **Detectable neutralization titers in stool**  **2 weeks after 1^st^ dose of nOPV2**  **(N=42/110)** | | | |  | **Detectable neutralization titers in stool**  **4 weeks after 1^st^ dose of nOPV2**  **(N=61/110)** | | | |
| --- | --- | --- | --- | --- | --- | --- | --- | --- | --- |
| Baseline log_2_ neutralization titers in serum | **n/N (%)** | **OR** | **95% CI** | ***P*** |  | **n/N (%)** | **OR** | **95% CI** | ***P*** |
| High (6.8-10.5) | 8/35 (22.9%) | Ref | Ref | -- |  | 19/35 (54.3%) | Ref | Ref | -- |
| Medium (5.1-6.5) | 12/34 (35.3%) | 1.8 | 0.6 - 5.3 | 0.26 |  | 16/34 (47.1%) | 0.7 | 0.3 - 1.9 | 0.55 |
| Low (2.5-4.8) | 22/41 (53.7%) | 3.9 | 1.4 - 10.6 | **0.008** |  | 26/41 (63.4%) | 1.5 | 0.6 - 3.7 | 0.42 |

**Supplementary Table 4. Poliovirus type 1 (PV1)- and type 3 (PV3)–specific intestinal mucosal responses following administration of novel type 2 oral poliovirus vaccine (nOPV2) or a placebo.**

*P* values are from Pearson’s chi-squared or Mann-Whitney U tests. Neutralization titers ≥16 were considered a positive neutralizing activity. Abbreviations: IgA, Immunoglobulin A; MFI, median fluorescence intensity; IQR, interquartile range.

*n=109 in week 6 in the nOPV2 group.

|  | Week | nOPV2 (N=110*)  median (IQR)  n/N(%) | Placebo (N=105)  median (IQR)  n/N(%) | *P* value |
| --- | --- | --- | --- | --- |
| Positive PV1 neutralizing activity | Baseline | 1/110 (0.9%) | 2/105 (1.9%) | 0.53 |
|  | 2 | 3/110 (2.7%) | 0/105 (0%) | 0.09 |
|  | 4 | 4/110 (3.6%) | 1/105 (1.0%) | 0.19 |
|  | 6 | 1/109 (0.9%) | 0/105 (0%) | 0.33 |
|  | 8 | 1/110 (0.9%) | 1/105 (1.0%) | 0.97 |
| PV1 IgA MFI | Baseline | 0.4 (0.4-0.4) | 0.4 (0.4-0.4) | 0.56 |
|  | 2 | 431.3 (101.3-1301.8) | 316.3 (0.4-1142.3) | 0.20 |
|  | 4 | 156.8 (0.4-728.3) | 271.3 (11.3-749.3) | 0.78 |
|  | 6 | 25.8 (0.4-240.8) | 2.8 (0.4-202.3) | 0.59 |
|  | 8 | 150.6 (0.4-514.8) | 131.3 (0.4-488.8) | 0.60 |
| PV1 IgG MFI | Baseline | 0.6 (0.6-93.8) | 27.3 (0.6-112.3) | 0.14 |
|  | 2 | 0.6 (0.6-0.6) | 0.6 (0.6-0.6) | 0.27 |
|  | 4 | 0.6 (0.6-0.6) | 0.6 (0.6-0.6) | 0.64 |
|  | 6 | 130.3 (3.3-249.3) | 106.3 (1.3-200.8) | 0.38 |
|  | 8 | 107.5 (0.6-228.3) | 126.3 (0.6-265.3) | 0.30 |
| Positive PV3 neutralizing activity | Baseline | 2/110 (1.8%) | 1/105 (1.0%) | 0.59 |
|  | 2 | 2/110 (1.8%) | 4/105 (3.8%) | 0.38 |
|  | 4 | 2/110 (1.8%) | 3/105 (2.9%) | 0.61 |
|  | 6 | 1/109 (0.9%) | 1/105 (1.0%) | 0.98 |
|  | 8 | 1/110 (0.9%) | 3/105 (2.9%) | 0.29 |
| PV3 IgA MFI | Baseline | 1.1 (1.1-23.6) | 1.1 (1.1-28.6) | 0.74 |
|  | 2 | 526.9 (242.6-1350.1) | 342.1 (139.1-1160.6) | 0.04 |
|  | 4 | 303.9 (119.1-691.6) | 353.1 (108.6-776.6) | 0.88 |
|  | 6 | 121.1 (30.6-308.1) | 101.6 (31.1-271.6) | 0.73 |
|  | 8 | 210.4 (86.1-542.1) | 240.1 (76.1-560.6) | 0.87 |
| PV3 IgG MFI | Baseline | 0.5 (0.5-102.5) | 0.5 (0.5-137.0) | 0.14 |
|  | 2 | 0.5 (0.5-0.5) | 0.5 (0.5-0.5) | 0.46 |
|  | 4 | 0.5 (0.5-2.5) | 0.5 (0.5-0.5) | 0.25 |
|  | 6 | 111.5 (0.5-339.0) | 101.5 (0.5-276.5) | 0.87 |
|  | 8 | 126.0 (0.5-266.5) | 86.0 (0.5-233.5) | 0.36 |

**Supplementary Table 5. Poliovirus type 2 (PV2)–specific intestinal detectable neutralization titers following administration of novel type 2 oral poliovirus vaccine (nOPV2) or placebo.**

*P* values are from Pearson’s chi-squared tests. Neutralization titers ≥4 were considered detectable.

|  | Week | nOPV2 (N=110)  n/N (%) | Placebo (N=105)  n/N (%) | *P* value |
| --- | --- | --- | --- | --- |
| Detectable PV2 neutralization titers | Baseline | 6/110 (5.5%) | 7/105 (6.7%) | 0.71 |
|  | 2 | 42/110 (38.2%) | 17/105 (16.2%) | **<0.0001** |
|  | 4 | 61/110 (55.5%) | 13/105 (12.4%) | **<0.0001** |
|  | 6 | 96/109 (88.1%) | 20/105 (19.1%) | **<0.0001** |
|  | 8 | 106/110 (96.4%) | 17/105 (16.2%) | **<0.0001** |

**Supplementary Table 6. Poliovirus type2 (PV2)–specific intestinal mucosal responses following administration of novel type 2 oral poliovirus vaccine (nOPV2) stratified by i/ sex, ii/ BCG received at birth.** *P* values are from Pearson’s chi-squared or Mann-Whitney U tests. Neutralization titers ≥16 were considered a positive neutralizing activity. Abbreviations: BCG, Bacillus Calmette-Guérin; IgA, Immunoglobulin A; MFI, median fluorescence intensity; IQR, interquartile range.

*n=53 week 6 ** n=65 week 6

|  | Week | Male (N=53*)  median (IQR) n/N (%) | Female (N=57)  median (IQR) n/N (%) | *P* value | BCG at birth (N=44)  median (IQR) n/N (%) | No BCG at birth (N=66**)  median (IQR) n/N (%) | *P* value |
| --- | --- | --- | --- | --- | --- | --- | --- |
| Positive PV2 neutralizing activity | Baseline | 1/53 (1.9%) | 1/57 (1.8%) | 0.96 | 0/44 (0%) | 2/66 (3.0%) | 0.24 |
|  | 2 | 14/53 (26.4%) | 17/57 (29.8%) | 0.70 | 14/44 (31.8%) | 17/66 (25.8%) | 0.49 |
|  | 4 | 25/53 (47.2%) | 32/57 (56.1%) | 0.35 | 24/44 (54.6%) | 33/66 (50.0%) | 0.64 |
|  | 6 | 39/52 (75.0%) | 44/57 (77.2%) | 0.78 | 33/44 (75.0%) | 50/65 (76.9%) | 0.82 |
|  | 8 | 48/53 (90.6%) | 51/57 (89.5%) | 0.85 | 40/44 (90.9%) | 59/66 (89.4%) | 0.80 |
| PV2 neutralization titer | Baseline | 2 (2-2) | 2 (2-2) | 0.95 | 2 (2-2) | 2 (2-2) | 0.66 |
|  | 2 | 2 (2-19.2) | 2 (2-38.1) | 0.83 | 2 (2-38.1) | 2 (2-18.3) | 0.43 |
|  | 4 | 5.9 (2-400.7) | 40.0 (2-261.4) | 0.83 | 22.8 (2-406.4) | 26.1 (2-261.4) | 0.76 |
|  | 6 | 158.1 (16.9-459.4) | 174.2 (21.2-420.9) | 0.99 | 170.0 (16.9-482.8) | 165.9 (21.2-400.7) | 0.87 |
|  | 8 | 442.0 (248.9-1336.1) | 406.4 (174.2-731.7) | 0.11 | 412.6 (170.4-949.2) | 442.0 (211.9-995.7) | 0.53 |
| PV2 IgA MFI | Baseline | 9.6 (0.1-48.1) | 8.1 (0.1-52.1) | 0.94 | 14.6 (0.1-60.1) | 7.9 (0.1-48.1) | 0.33 |
|  | 2 | 647.1 (280.6-1753.1) | 655.1 (300.6-1379.1) | 0.74 | 673.6 (314.6-1565.4) | 651.1 (280.6-1409.1) | 0.71 |
|  | 4 | 737.1 (228.6-1417.6) | 682.1 (234.4-1608.4) | 0.82 | 786.6 (207.1-1417.6) | 691.4 (263.1-1979.6) | 0.53 |
|  | 6 | 520.1 (245.6-1309.6) | 573.1 (208.1-956.1) | 0.80 | 496.4 (173.9-1100.4) | 561.1 (255.6-1209.6) | 0.47 |
|  | 8 | 1187.1 (842.1-2421.6) | 1441.6 (625.6-2021.1) | 0.67 | 1179.1 (597.4-2293.4) | 1290.6 (842.1-1957.1) | 0.94 |

**Supplementary Figure 3. Correlation of total immunoglobulin A (IgA, µg/ml), poliovirus serotype-specific (PV1, PV2, PV3) median fluorescence intensities (MFIs) IgA, and lactoferrin levels (ng/ml) in stool at baseline (days 0-3; before vaccination) stratified by age at study enrollment (in days).** Colors indicate the age at enrollment. Both randomization groups were included.
